# Proteogenomics for pediatric brain cancer

**Published:** 2021-09-01

**Authors:** Margaret SIMONIAN

**Affiliations:** University of Californian Los Angeles, David Geffen School of Medicine, Los Angeles, 90095, USA

**Keywords:** Proteogenomics, Brain cancer, Targeted therapy

## Abstract

Pediatric central nervous system tumors are the most common tumors in children, it constitute 15%–20% of all malignancies in children and are the leading cause of cancer related deaths in children. Proteogenomics is an emerging field of biological research that utilizes a combination of proteomics, genomics, and transcriptomics to aid in the discovery and identification of biomarkers for diagnosis and therapeutic purposes. Integrative proteogenomics analysis of pediatric tumors identified underlying biological processes and potential treatments as well as the functional effects of somatic mutations and copy number variation driving tumorigenesis.

## Introduction

Pediatric brain tumors are the leading cause of cancer related deaths in children. Pediatric brain tumors are masses of abnormal cells that occur in a child’s brain, some are benign and some are malignant. Paediatric brain tumors treatment is different than adult brain tumors treatment and it depends on the type, size, and the location of the tumor within the brain, as well as the child’s age and general health (Pollack *et al*., 2006). The four main pediatric brain tumors are medulloblastoma, ependymoma, high-grade glioma and low-grade glioma (Zhukova *et al*., 2019).

### Current treatment options

#### Surgery

Surgical removal is performed when the tumor is small in size and easily accessible for complete surgical removal. Although it carries risks especially when tumors cannot be separated from surrounding tissue or when they are located near sensitive areas in the brain (Pollack *et al*., 2006).

#### Radiation therapy (RT)

Such as X-rays or proton beam therapy that delivers high targeted doses of radiation to brain tumors, while minimizing radiation exposure to surrounding healthy tissues. RT has undergone comprehensive changes in recent decades to maximize the therapeutic benefit between efficacy and toxicity, such as the development of IMRT, VMAT and PBRT radiation therapy (de Nunzio and Yock, 2020).

#### Radiosurgery

Stereotactic radiosurgery delivers multiple highly focused beam of radiation Gamma Knife or linear accelerator treatment to destroy the tumor in a small area. The point where all the beams meet receives the highest dose of radiation while minimizing the radiation exposure to surrounding tissues. Radiosurgery in children is important for treating unresectable tumors or recurrent tumors that have received prior radiotherapy (de Nunzio and Yock, 2020).

#### Chemotherapy

Chemotherapy uses drugs to destroy cancer cells, normally injected through the vein (intravenous chemotherapy). The option of chemotherapy drugs depends on the type of cancer (Zhukova *et al*., 2019).

#### Targeted drug therapy

**FIGURE 1.**
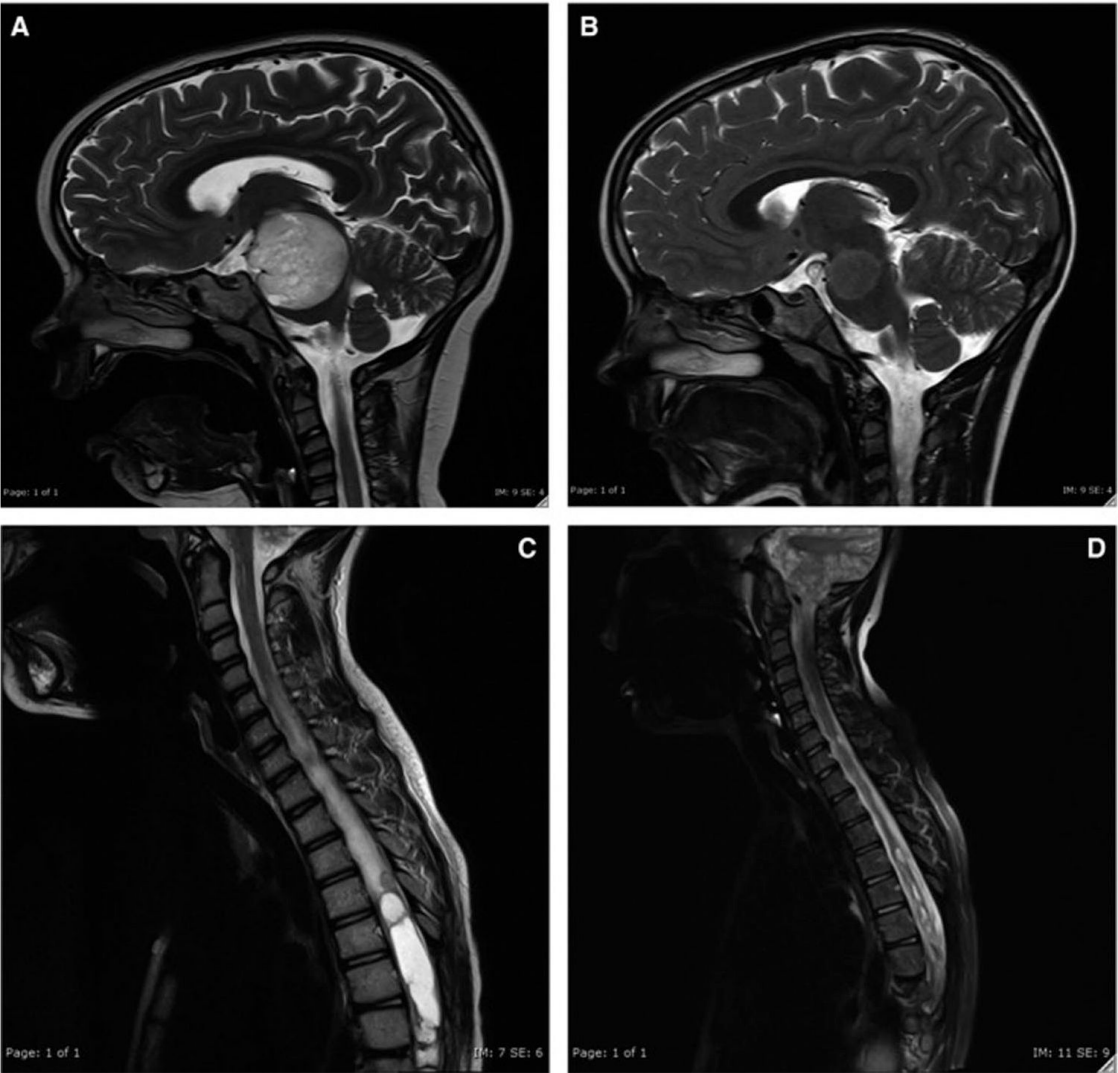
Radiological response to bevacizumab therapy; MRI imaging at baseline PLGG (A) and PLGG10 (C) and 15 mo after completion of therapy PLGG8 (B) and after 6 mo of therapy PLGG10 (D) (Zhukova *et al*., 2019).

Targeted therapy *uses* drugs to target specific genes and proteins that are involved in the growth and survival of *cancer* cells, one example is bevacizumab that is used to treat low-grade gliomas (Zhukova *et al*., 2019) (Fig. 1). Genomic techniques started to elucidate the pathogenesis of many pediatric brain tumors, however, there are some challenges that limit the translation of these findings into therapy (Kumar *et al*., 2018; Chalmers *et al*., 2017; Grobner *et al*., 2018; Northcott *et al*., 2017). The majority of pediatric brain tumors resist genomic targeted treatments. In addition many pediatric brain tumors are characterized by deviant epigenetic landscapes, and there is no effective way to specifically target these changes (Capper *et al*., 2018).

#### Proteogenomics

Recently quantitative mass spectrometry analyses have advanced, adding a quantitative proteomics approach to a primarily genomics based biological understanding of cancers; this gave rise to a new emerging technology called “Proteogenomics” (Dou *et al*., 2020; Simonian *et al*., 2017; Zhang *et al*., 2016). Proteogenomics utilizes a combination of proteomics, genomics, and transcriptomics to support the discovery and identification of novel peptides from mass spectrometry based data (Zhang *et al*., 2016). Hence, analysis of integrated proteogenomics datasets has the potential to aid with identification of new therapeutic targets and avenues.

Proteogenomics recently have been used in many cancer studies, such as breast, colon and liver cancers to better understand tumor biology and provide therapeutic targets (Krug *et al*., 2020; Vasaikar *et al*., 2019; Gao *et al*., 2019). In 2020, Petralia *et al*. (2020), conducted the first comprehensive proteogenomic study across major histological types of pediatric brain cancer. The analysis included whole-genome sequencing RNA sequencing (transcriptomics), proteomics (LC-MS, MRM, TMT) and phosphoproteomics characterization, of 218 tumors across 7 histological types of childhood brain cancer: low-grade glioma (LGG), high-grade glioma (HGG), ependymoma (EP), craniopharyngioma (CP) medulloblastoma (MB), ganglioglioma, and atypical teratoid rhabdoid tumor (ATRT). This was the first large-scale proteogenomics analysis across traditional histological boundaries to reveal foundational pediatric brain tumor biology and reveal rational treatment options (Petralia *et al*., 2020) Fig. 2.

The Proteomics data identified familiar biological patterns across histological samples, suggesting that treatments used for one histological type may be applied effectively to other tumors with similar proteomics characteristics. The data further revealed functional effects of somatic mutations and copy number variations not visible in the transcriptomics data (Petralia *et al*., 2020).

Kinase-substrate association and co-expression network analysis also identified important tumorigenesis mechanisms. Investigation of kinase activity based on phosphoproteomics showed that protein abundance can be reduced in active signaling pathways (e.g., ERK1/3 in CP), which could happen because of feedback loops in many complicated regulatory processes, suggesting the important role of phosphoproteomics data in characterizing pathway activities (Petralia *et al*., 2020).

The proteomics/phosphoproteomics data clustering analyses uncovered two apparent subgroups of pediatric CP, with one subgroup showing similar proteomics/phosphoproteomics characteristics as pediatric LGG *BRAF*^V600E^ tumors. This observation suggests potential use of MEK/MAPK inhibitors in a subset of pediatric CP, which currently has no effective chemotherapy options. The existence of these two subgroups of CP, was not evident from RNA data. Additionally they observed a disagreement between RNA and protein abundance in other histologies, such as EP and LGG (Gao *et al*., 2019). The low to moderate RNA-protein correlation in the study was consistent with other large-scale proteogenomics projects studies (Dou *et al*., 2020; Clark *et al*., 2019; Gillette *et al*., 2020). Multiple factors, such as protein turnover and selective translation, may have contributed to the low correlation between RNA and protein abundance. Furthermore, more aggressive tumors showed increased protein-RNA correlation, this occurrence was observed among multiple cancer proteogenomics studies (Dou *et al*., 2020; Clark *et al*., 2019). One possible explanation is that aggressive tumors often have high proliferation, and the boosted translation activities in highly proliferative tumor cells result in more correlated RNA and protein signals. Hence, studying the proteome revealed insights that were not evident from RNA-based analysis alone (Petralia *et al*., 2020).

Pediatric tumors normally have fewer genetic variation and somatic mutations compared to adult tumors (Grobner *et al*., 2018; Sweet-Cordero and Biegel, 2019; Barkhoudarian *et al*., 2016). However few recurrent DNA alterations were observed in the above study. LGG tumors with BRAF^*V600E*^ mutation had significantly downregulated BRAF protein abundance compared with BRAF^*WT*^ LGG tumors where in fact the reduction was not significant at the transcript level. CTNNB1 mutation resulted in elevated protein/RNA levels among CP samples, whereas NF1 mutation resulted in down regulation of associated proteins and transcripts in HGG. SMARCB1 RNA/protein were also significantly downregulated in ATRT samples and the downregulation was the result of different types of DNA alterations, including mutation, deletion, and copy-neutral loss of heterozygosity (Petralia *et al*., 2020).

## Conclusion

These studies demonstrated the ability of proteomics, phosphoproteomics, and kinase activity scores to elucidate active signaling processes within tumors types. Applying these capabilities in a clinical trial study could reveal valuable information regarding the biology of individual tumors that respond to a given therapy. Once signature proteomics targets are identified, MRM (multiple reaction monitoring) signatures can be developed to identify individuals in real time whose tumors display particular biologics hence, may respond to a treatment. Similar histological tumors are usually treated differently in pediatric and adult patients, although they often differ in their genomic features, this study has shown that they do not always drive biology. Future proteomics work is needed to cross examine tumors whose incidence compass a large age range to answer questions regarding how biology changes across the spectrum and if treatments can be rearranged for maximum benefit. A future study gathering larger group of the less common brain tumor types would also be instructive in identifying the biology that is unique to those tumors.

## Figures and Tables

**FIGURE 2.**
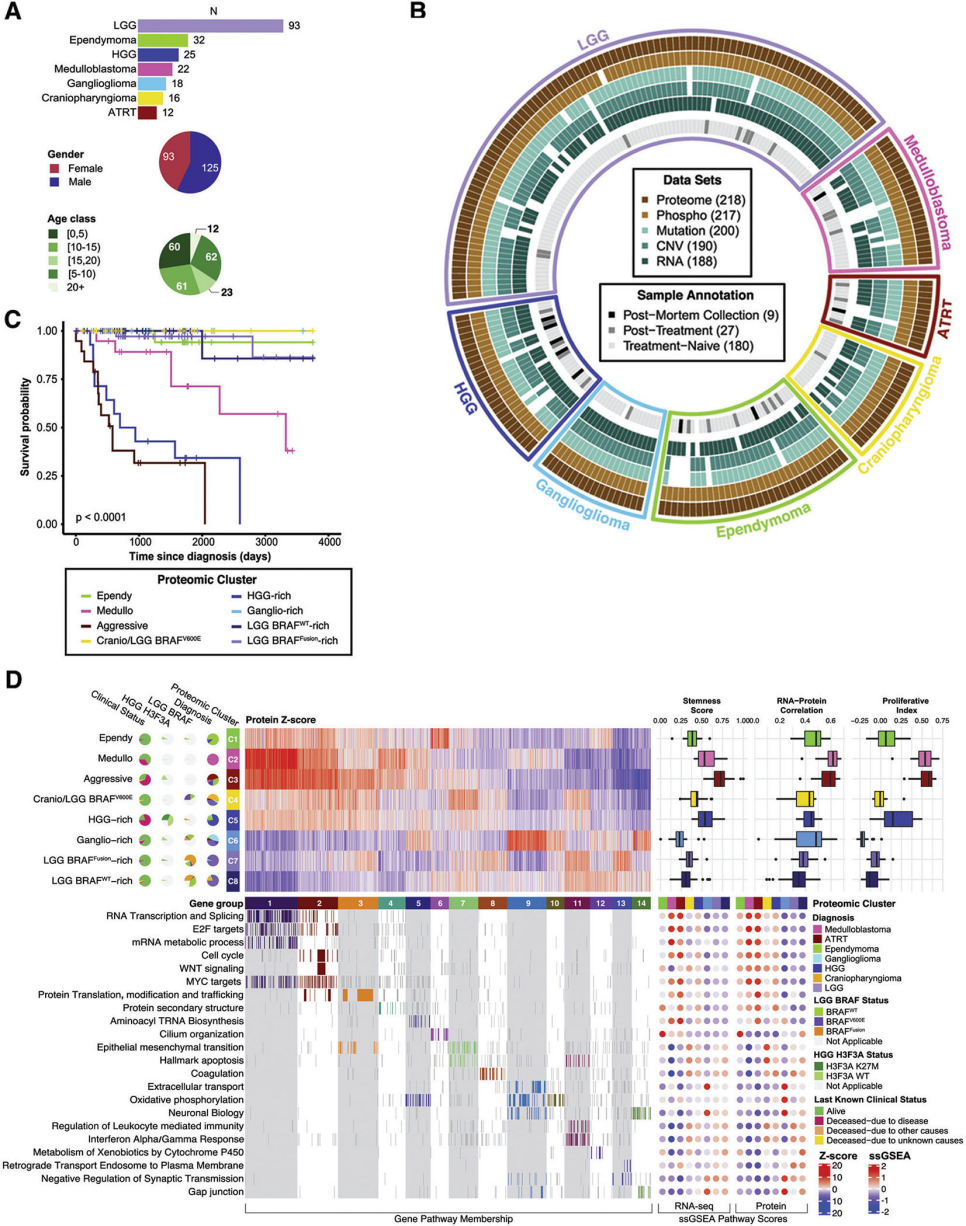

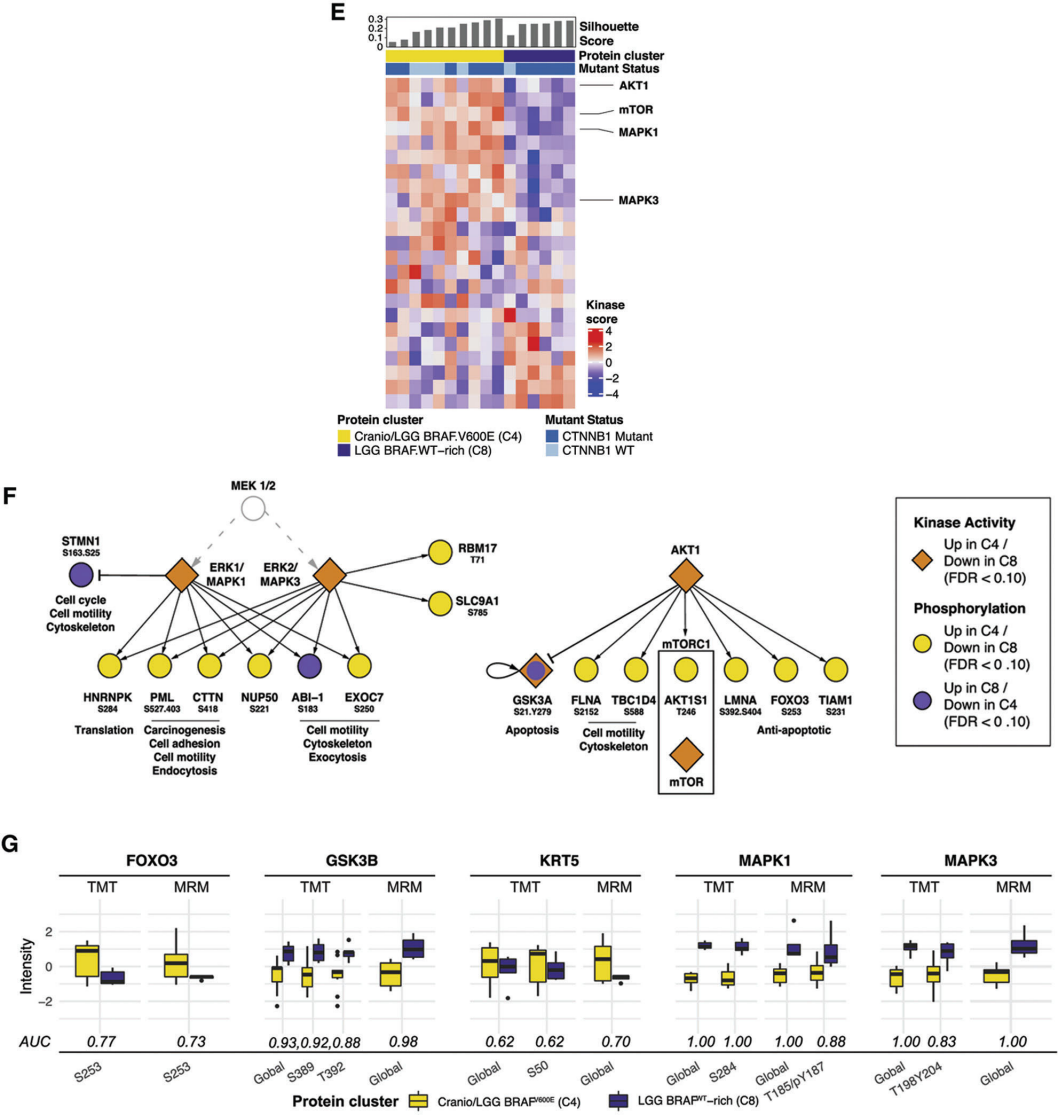
Proteomics clustering of pediatric brain tumors showing, summary of the brain tumor cohort, presence of omics datasets for each of the 218 tumor samples, Kaplan-Meier curves for overall survival (OS) of patients stratified by proteomic cluster, proteomic clusters and differentially expressed proteins allocated to 14 gene groups, heatmap of kinase activity scores for CP tumors and diagram showing differences between C4 and C8 CP tumors in terms of phosphorylation abundance and kinase activity for AKT and ERK1/2 signaling members. For full figure description see (Petralia *et al*., 2020).
